# The Effect of Dietary Inorganic Nitrate Supplementation on Cardiac Function during Submaximal Exercise in Men with Heart Failure with Reduced Ejection Fraction (HFrEF): A Pilot Study

**DOI:** 10.3390/nu12072132

**Published:** 2020-07-17

**Authors:** Mary N. Woessner, Itamar Levinger, Jason D. Allen, Luke C. McIlvenna, Christopher Neil

**Affiliations:** 1 Institute for Health and Sport (IHES), Victoria University, Melbourne, VIC 3011, Australia; Itamar.Levinger@vu.edu.au (I.L.); ja6af@virginia.edu (J.D.A.); luke.mcilvenna@live.vu.edu.au (L.C.M.); christopher.neil@unimelb.edu.au (C.N.); 2 Australian Institute for Musculoskeletal Science (AIMSS), Western Health, St Albans, VIC 3021, Australia; 3 Department of Kinesiology & Division of Cardiovascular Medicine, University of Virginia, Charlottesville, VA 22903, USA; 4 Department of Medicine-Western Health, University of Melbourne, St Albans, VIC 3021, Australia; 5 Western Health Chronic Disease Alliance, University of Melbourne, St Albans, VIC 3021 Australia

**Keywords:** nitric oxide, cardiovascular disease, heart failure, cardiac performance

## Abstract

Heart failure with reduced ejection fraction (HFrEF) is a common end point for patients with coronary artery disease and it is characterized by exercise intolerance due, in part, to a reduction in cardiac output. Nitric oxide (NO) plays a vital role in cardiac function and patients with HFrEF have been identified as having reduced vascular NO. This pilot study aimed to investigate if nitrate supplementation could improve cardiac measures during acute, submaximal exercise. Five male participants (61 ± 3 years) with HFrEF (EF 32 ± 2.2%) completed this pilot study. All participants supplemented with inorganic nitrate (beetroot juice) or a nitrate-depleted placebo for ~13 days prior to testing. Participants completed a three-stage submaximal exercise protocol on a recumbent cycle ergometer with simultaneous echocardiography for calculation of cardiac output (Q), stroke volume (SV), and total peripheral resistance (TPR). Heart rate and blood pressure were measured at rest and during each stage. Both plasma nitrate (mean = ~1028%, *p* = 0.004) and nitrite (mean = ~109%, *p* = 0.01) increased following supplementation. There were no differences between interventions at rest, but the percent change in SV and Q from rest to stage two and stage three of exercise was higher following nitrate supplementation (all *p* > 0.05, ES > 0.8). Both interventions showed decreases in TPR during exercise, but the percent reduction TPR in stages two and three was greater following nitrate supplementation (*p* = 0.09, ES = 0.98 and *p* = 0.14, ES = 0.82, respectively). There were clinically relevant increases in cardiac function during exercise following supplementation with nitrate. The findings from this pilot study warrant further investigation in larger clinical trials.

## 1. Introduction

Individuals with coronary artery disease are characterised by extensive damage to the vasculature which can lead to plaque formation, a myocardial infarct, and ultimately reach an endpoint diagnosis of heart failure with reduced ejection fraction (HFrEF) [1]. Patients with HFrEF are characterised by lower aerobic capacity (VO_2peak_) and increased fatigue during sub maximal and maximal exercise. While changes in the peripheral tissues contribute to the reduced exercise capacity in HFrEF, the primary initiator of clinical disability is the significant reduction in cardiac output (Q) at rest and during exercise [2]. Thus, therapeutic interventions that can improve cardiac function are likely to contribute to better clinical outcomes in this population.

**Figure 1:**
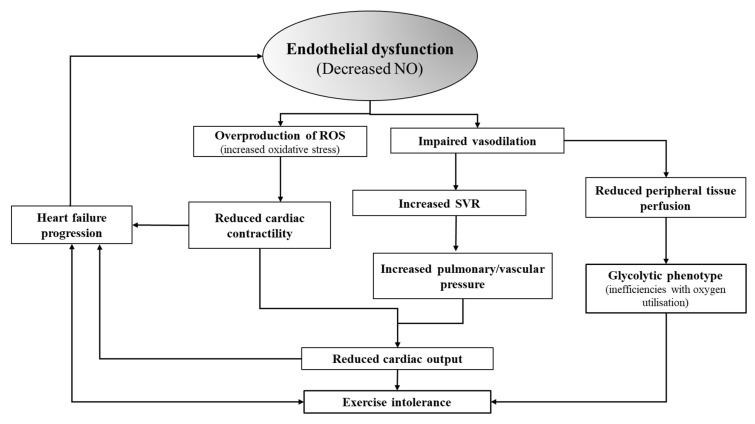
Role of NO in central and peripheral dysfunctions in HFrEF.

Nitric oxide (NO) is a ubiquitous free radical signalling molecule influencing a variety of physiological processes. In the vasculature it functions as a potent vasodilator synthesized by endothelial nitric oxide synthase (eNOS) [3,4]. It’s vasodilatory role is especially critical during exercise when there is an increased demand for oxygenated blood in the working tissues [5]. While previous studies have highlighted the critical role of NO primarily within the peripheral vasculature, there is some evidence that NO can mediate coronary vasodilation and potentially cardiac contractility [6]. However, individuals with HFrEF have an inability to endogenously upregulate NO. The lack of NO results in less vasodilation, increased systemic vascular resistance and increased pressures in both the vascular and pulmonary systems (Figure 1). These hemodynamic disturbances can acutely reduce cardiac output and exercise tolerance and can eventually lead a progression of the syndrome [7,8,9]. Therefore, an intervention that can increase NO bioavailability could potentially improve cardiac function in individuals with HFrEF [9,10].

Endothelial dysfunction (reduced NO) is both a precursor and progressor of the CHF syndrome itself through the negative impacts of increased oxidative stress on cardiac contractility and the effects of vasoconstriction on increasing resistance and pressure in the pulmonary and vascular system. Abbreviations: NO- nitric oxide, Q- cardiac output, ROS- reactive oxygen species.

Inorganic nitrate supplementation is an established intervention for increasing NO bioavailability in both healthy and diseased populations [11,12]. While previous studies have demonstrated improvements in exercise performance and vascular outcomes in diverse clinical cohorts, the majority of studies in HFrEF have indicated that supplementation has a minimal effect [13,14,15,16] However, evidence in HFpEF suggests the potential for nitrate (and nitrite) supplementation to improve systemic vascular resistance (SVR) and cardiac output (CO) [10,17]. To our knowledge, no study to date has examined the effects of supplementation on measures of cardiac function during exercise. The aim of this pilot study was to test the hypothesis that short-term inorganic nitrate supplementation could improve cardiac function during submaximal exercise in male patients with HFrEF.

## 2. Methods

### 2.1. Study Design

The exercise echocardiograph testing was conducted as an optional pilot study component of a larger clinical trial for which the protocol was previously published [18]. In brief, the study was a randomized, placebo-controlled, double blind, crossover study. This paper focuses on the results of the cardiac response to submaximal exercise visit. It was approved by the Melbourne Health Ethics Committee, has mirror approval by Victoria University Ethics Committees and has been registered in the Australian New Zealand Clinical Trials Registry [ACTRN12615000906550].

### 2.2. Recruitment Consent and Screening

Participants were recruited from the heart failure clinic of Sunshine Hospital. The full inclusion/exclusion criteria have been previously published, but in brief individuals with an EF% < 40, no recent (within six weeks) overnight hospital stays, and who were clinically stable were included [18].Interested and eligible individuals were invited to sign an informed consent. The process of obtaining written consent was conducted in accordance with the Declaration of Helsinki. Following consent, participants underwent a medical screening including a cardiopulmonary exercise test (CPX). If participants screened into the study, they were then randomized to consume 210 mL/day of either a nitrate rich beetroot juice (BTR) (~16 mmol) or a nitrate-depleted beetroot juice (PL) (>0.1 mmol) for approximately 13 ± 5 days (due to availability of cardiac lab and cardiologist). Participants then completed the submaximal exercise test with simultaneous echocardiography (described below). There was a two-week washout period between treatment arms.

### 2.3. Plasma Nitrate/Nitrite Analysis

Blood samples were taken at the mid-point of supplementation (~day 6) during the CPET visit of the main study. Sample collections were timed to be taken at 2.5 h post-ingestion of the morning dose of BTR or PL. On collection samples were centrifuged for 3 min at 5000 rpm, then plasma was snap frozen in liquid nitrogen and stored at −80 until analysis. All NO metabolite concentrations were measured (within 10 min of defrosting) by gas-phase chemiluminescence using a NOA 280i Ionics/Sievers nitric oxide analyzer, as per manufacturer’s instructions (Sievers Instruments, Boulder, CO, USA) [19].

### 2.4. Submaximal Exercise Echocardiography

All participants were asked to refrain from strenuous exercise and to avoid alcohol consumption for the 24 h before their scheduled testing visit. They were also asked to not consume caffeine on the day of testing visit. The timing of the testing visit was kept consistent for each participant to minimize the effect of individual diurnal variations in blood pressure and cardiac responses. Additionally, all imaging was performed by the same sonographer.

Participants were connected to a 12-lead ECG and fitted on an echo-compatible recumbent cycle ergometer (Vivid 7 echocardiographic machine, GE, Milwaukee, WI, USA). The recumbent cycle was specifically designed for exercise with simultaneous echocardiography. It positions the individual in a semi-supine and tilted position during exercise which allows for a more accurate assessment of cardiac function under stress. While not fully recumbent, the positioning could facilitate an increase in venous return.

The protocol consisted of three discontinuous stages of individualized incremental light to moderate intensity workloads (15–25 W, 25–40 W and 35–60 W) chosen to elicit BORG rating of perceived exertion (RPE) values of approximately 9 (very light), 11 (fairly light), and 13 (somewhat hard). There was a five-minute break between each stage to allow for recovery. Images and videos were captured approximately two and a half minutes into each five-minute stage. The exercise for each stage was ceased when the imaging was complete. Heart rate was continuously monitored, and blood pressure was recorded at rest, three minutes into each stage and hallway through each rest period. RPE was captured in the final 10 s before the end of each stage.

Cardiac output (Q) and stroke volume (SV) were derived using Doppler Velocity Time Integral according to the Huntsman method [20]. This method utilizes measures of left ventricular outflow tract diameter (LVOT) and LVOT sub valvular velocity time integral (VTI) to estimate Q and SV utilizing the following equations:

$$SV=\pi \times \left(\frac{LVOT}{2}\right)2\;\times LVOT\;VTI$$

$$CO=\frac{SV\;\times \;HR}{1000}$$

Mean arterial pressure (MAP) was calculated as:

$$MAP=\left[\frac{\left(SBP-DBP\right)}{3}\right]+DBP$$

Total peripheral vascular resistance (TPR) was calculated from the following formula [21]:

$$TPR\;\;\left(\mathrm{dyne}\cdot \sec\cdot {\mathrm{cm}}^{-5}\right)=\frac{80\times MAP\;\left(\mathrm{mmHg}\right)}{Q\;\left(L\cdot \min^{-1}\right)}$$

### 2.5. Statistical Analysis

All statistical analysis was conducted using the Statistical Package for the Social Sciences (version 22, SPSS Inc. Chicago, IL, USA). Analysis of the differences in plasma nitrate and nitrite values between interventions were completed with paired samples t-test (two-sided, with *p* < 0.05 considered statistically significant). The calculation of the magnitude of change using Cohen’s effect size (ES) was used as the analysis for the primary and secondary outcome variables. This parameter has been found to be representative of the clinical relevance of an interventional change than the *p*-value alone [22]. ES change is quantified as small, 0.2; moderate. 0.5; large 0.8 or very large 1.2 [23]. In addition to the ES calculations, secondary analysis included a two-way repeated measures ANOVA to assess the differences in change scores (from rest to each exercise stage) in cardiac function between the placebo and nitrate interventions. GraphPad Prism Version 7.00 for Windows (GraphPad Software, La Jolla, CA, USA; www.graphpad.com) was utilized for the creation of all graphs and figures. All results are presented as mean ± standard error.

## 3. Results

Patient characteristics are reported in Table 1. There were no significant differences between the number of days of dosing in BTR or PL intervention (13 ± 2 days versus 12 ± 3 days, *p* = 0.74). There were no differences in the exercise duration of stages two or and three between treatments, however, due to image acquisition issues, there stage one of exercise lasted approximately 20 s longer on average in the nitrate arm (nitrate 386 ± 23 s, placebo 364 ± 20 s, *p* < 0.04).

**Figure 2:**
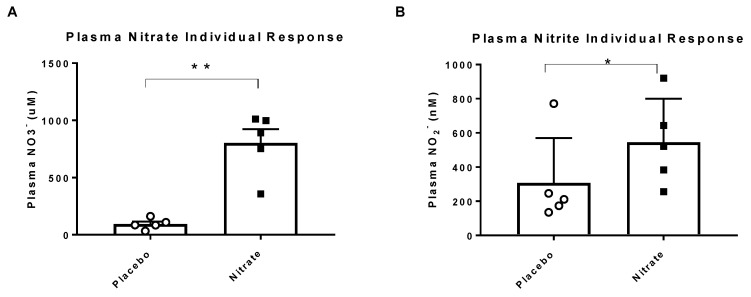
Individual and mean responses of plasma nitrate (**A**) and plasma nitrite (**B**). Black boxes are the nitrate treatment and the white circles represent placebo treatment. Abbreviations: NO_3-_, nitrate, NO_2-_, nitrite, * indicates significance at *p* < 0.05, ** indicates significance at *p* < 0.01.

All five participants had higher concentrations of plasma nitrate (1028 ± 418%, *p* < 0.05) and nitrite (109 ± 30%, *p* < 0.01) following nitrate supplementation as compared to placebo treatment (Figure 2).

At rest, there were no differences between the placebo and nitrate interventions in Q, SV or TPR (Table 2). When examining absolute values at each stage, there were medium to very large ES for the differences between placebo and nitrate in stage two and stage three for Q, SV and TPR.

**Figure 3:**
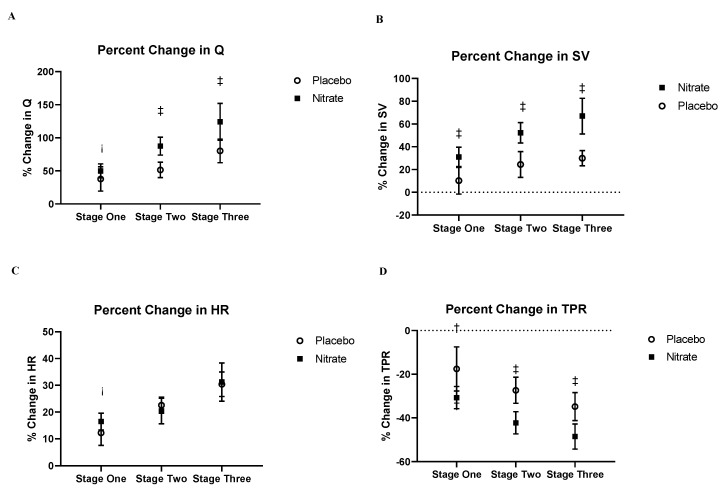
Resting and submaximal exercise responses of (**A**) cardiac output, (**B**) stroke volume, (**C**) heart rate, (**D**) total peripheral resistance.

The percent change in SV and Q from rest to stage two and stage three was higher (all ES > 0.80) following nitrate supplementation as compared to placebo (Figure 3). These elevations in SV and Q following nitrate supplementation were likely mediated by the greater reduction (ES > 0.8 for stage two and stage three) in TPR following nitrate supplementation as compared to the placebo intervention (Figure 3D).

The data represents the percent change of each variable between rest and each exercise intensity. The difference in the percent change increase in Q and SV between the nitrate and placebo group had a large ES in stages two and three of exercise (A and B), with the nitrate group having an increased Q and SV relative to placebo. The difference in the percent change reduction in TPR between placebo and nitrate interventions had medium and large ES changes during stages one, two and three respectively. There was only a small ES for the difference in percent change of HR at stage one between interventions. Cohen’s D effect sizes of the change between interventions is indicated as follows: small (>0.3) ¡, medium (>0.5) ES-†, large (>0.8) ES-‡. Abbreviations: Q, cardiac output, SV, stroke volume, TPR, total peripheral resistance, HR, heart rate.

In support of this finding, there were medium to large Cohen’s D ES for the difference in absolute values for diastolic blood pressure (DBP) and mean arterial blood pressure (MAP) at all stages of exercise (Table 3).

## 4. Discussion

The key findings of this preliminary pilot study indicate that supplementation with nitrate may acutely improve Q in patients with HFrEF via concomitant reductions in vascular resistance in the peripheral tissues. Improvements in cardiac function following supplementation could have significant implications for this population, but future studies with larger sample sizes are required to confirm these results and to explore whether the effects can be maintained over a longer period.

Patients with CHF have a significant reduction in Q during exercise. This reduction is an established contributor to the hallmark symptom of CHF, reduced exercise tolerance [24]. As such, an intervention that can partially restore Q during exercise has significant implications for clinical wellbeing. Herein, we report that oral inorganic nitrate supplementation resulted in a greater increase in Q in two (out of three) stages of discontinuous incremental exercise. These differences were seen specifically during the higher intensity workloads, which is in line with previous work indicating the effects of nitrate supplementation are more pronounced in low oxygen conditions [13]. This finding is in agreement with an earlier study by Zamani et al. in which patients with HFpEF demonstrated increases in Q (measured with echocardiography) following a single acute dose of 12.9mmol inorganic nitrate [17]. The authors suggested that the change in Q was mediated by an increase in SV in response to reduced TPR. In HFrEF, the only previous study measuring Q found that nitrate supplementation did not affect Q, as measured by impedance cardiography [15]. Future studies should seek to measure TPR directly to confirm this finding.

Previous studies have indicated intra and inter-variations in plasma nitrite response in healthy and clinical cohorts [12]. In both the main study and in the present study, plasma nitrite increased significantly (109 ± 30%, *p* = 0.01) following nitrate supplementation, indicating that the supplementation was effective in this cohort [25]. Given that there is still no consensus on the optimal dosing amount or duration of nitrate supplementation, the higher and longer dose in the present study could have potentiated the effects of nitrate and explain the positive findings.

The study has some potential limitations. While recruitment was open to all individuals with HFrEF, the study consisted of only male participants, which limits the applicability of the findings to women. This pilot study utilized an indirect calculation of cardiac function and vascular resistance. Direct measurements require an invasive study design that was beyond the capability of this project. There is congruity between direct measures of cardiac function and echocardiograph measurements during exercise, but the use of direct measurement of cardiac function and structure should be considered in future trials [26,27]. Finally, while there were significant increases in plasma nitrite, the actual percentage change was still relatively small and could suggest an underlying defect in the nitrate reducing pathway of individuals with HFrEF.

In conclusion, we reported clinically relevant improvements in measures of cardiac function during submaximal exercise following inorganic nitrate supplementation in patients with HFrEF. The potential beneficial effect of nitrate supplementation on vascular resistance suggests that this intervention could be particularly beneficial in those individuals with underlying vascular disease.

## Figures and Tables

**Table 1:** Participant Demographics.

Variable	Value
Age, mean ± SEM, y	61 ± 3
Height, mean ± SEM, cm	172 ± 3.0
Mass, mean ± SEM, kg	91 ± 5.8
BMI	30.9 ± 2.0
VO_2peak_ mL/kg/min	17.2 ± 2.1
EF (%)	32 ± 2.2
Etiology	-
Ischemic	3
Non-Ischemic Dilated Cardiomyopathy	1
Idiopathic Heart Disease	1
NYHA Class, *n* (%)	-
Class I	2 (40)
Class II	3 (60)
Comorbidities, *n* (%)	-
Diabetic	3 (60)
HTN	1 (20)
Obese	4 (80)
Drug therapy, *n* (%)	-
Metformin	1 (20)
B-Blockers	5 (100)
ACE Inhibitor	2 (40)
Statin	3 (60)
Aspirin	4 (80)
Diuretics	3 (60)

Abbreviations: EF, ejection fraction; NYHA, New York Heart Association; COPD, chronic obstructive pulmonary disease; HTN, hypertension; ACE inhibitor- angiotensin-converting-enzyme inhibitor.

**Table 2:** Cardiac Function Response at Rest and During Exercise.

Variable	Supplementation	Rest	Stage 1	Stage 2	Stage 3
Q (L/min)	Placebo	3.6 ± 0.3	4.9 ± 0.5	5.3 ± 0.2	6.5 ± 0.7
-	Nitrate	3.5 ± 0.4	5.1 ± 0.4	6.4 ± 0.4 ^#^	7.5 ± 0.6 ^†^
SV (mL/beat)	Placebo	52.5 ± 5.3	57.1 ± 7.1	63.4 ± 3.9	67.7 ± 6.2
-	Nitrate	47.2 ± 3.6 ¡	61.4 ± 4.7	71.3 ± 5.0 ^‡^	78.6 ± 8.4 ^†^
TPR (A.U.)	Placebo	2034 ± 261.2	1629 ± 213.9	1419 ± 93.9	1301 ± 178.1
-	Nitrate	2053 ± 282.5	1370 ± 100.8 ¡	1152 ± 120.4 ^#^	998.4 ± 60.0 ^‡^

Absolute values at each stage are presented. There were no significant differences in any of the absolute values at rest or at any exercise intensities. Differences between absolute values for Q, SV and TPR at rest and each stage are indicated by ES. Cohen’s D ES of the difference between the interventions were indicated as: small (>0.3) ¡, medium (>0.5) ES-^†^, large (>0.8) ES-^‡^, very large (>1.2) ES-^#^.

**Table 3:** Blood Pressure Response at Rest and During Submaximal Exercise.

Variable	Supplementation	Rest	Stage 1	Stage 2	Stage 3
SBP	Placebo	116 ± 8	125 ± 2	126 ± 4	134 ± 8
	Nitrate	112 ± 6	124 ± 6	123 ± 7	136 ± 8
DBP	Placebo	74 ± 6	78 ± 8	78 ± 4	81 ± 5
	Nitrate	72 ± 7	67 ±5 ^†^	73 ± 4 ^‡^	71 ± 5 ^†^
MAP	Placebo	88 ± 5	93 ± 6	94 ± 3	99 ± 4
-	Nitrate	85 ± 6	86 ± 4 ^†^	89 ± 5 ^‡^	93 ± 6 ^†^

No differences were noted between interventions for SBP at rest or any of the exercise stages. The difference in DBP between interventions during stage two and three had large and medium ES, respectively. There were medium to large ES for the differences between interventions in MAP and RPP at all stages of exercise. All data is presented as mean ± SEM. Cohen’s D effect sizes of the difference between interventions are indicated as follows: medium (>0.5) effect size-†, large (>0.8) effect size-‡. Abbreviations: SBP, systolic blood pressure, DBP, diastolic blood pressure, MAP, mean arterial blood pressure.
